# Utilizing host endogenous microRNAs to negatively regulate the replication of porcine reproductive and respiratory syndrome virus in MARC-145 cells

**DOI:** 10.1371/journal.pone.0200029

**Published:** 2018-07-03

**Authors:** Liwei Li, Fei Gao, Hao Zheng, Yifeng Jiang, Wu Tong, Yanjun Zhou, Guangzhi Tong

**Affiliations:** 1 Shanghai Veterinary Research Institute, Chinese Academy of Agricultural Sciences, Shanghai, PR China; 2 Jiangsu Co-Innovation Center for the Prevention and Control of Important Animal Infectious Disease and Zoonose, Yangzhou University, Yangzhou, PR China; Chuo University, JAPAN

## Abstract

MicroRNAs (miRNAs) contribute to gene regulation at the post-transcriptional level and are capable of mRNA silencing by binding to target sites exhibiting high degrees of complementarity. Therefore, cloning host miRNA-recognition sequences into the genome of RNA viruses represents a rational strategy for manipulating viral replication. Here, we performed deep sequencing to obtain small-RNA (sRNA)-expression profiles from *in vitro*-cultured MARC-145 cells post infection with porcine reproductive and respiratory syndrome virus (PRRSV) and chose six candidate miRNAs of different abundance (miR-21, miR-140-3p, miR-185, miR-26a, miR-505, and miR-199a) for further study. Based on the full-length cDNA clone p7USC, we constructed a number of PRRSV mutants that provided complementary base-pairing target sites for the miRNAs in 3′ untranslated regions. Our results showed that all low- and moderate- abundant miRNA-target mutants showed similar growth properties, whereas the highest-abundant miRNA-target mutant blocked both viral transcription and replication. Discontinuous mutations in high-abundant miRNA-target sites subsequently recovered viral viability and propagation. These results demonstrated the copy number of endogenous miRNAs and the extent of sRNA complementarity were key factors to silence potential mRNA expression/translation, thereby determining PRRSV viability. Interestingly, the mutant containing miR-140-target sites (v140-t) showed strong suppression of viral replication from P1 to P3 *in vitro*, as shown by virus titer, plaque morphology, and qRT-PCR assays. To assess genetic stability, sequencing of v140-t (P1, P3, P5 and P10) revealed spontaneous mutations preferentially located among several nucleotides near the 3′ end of the insertion region and corresponding to the “seed region” of miR-140-3p, explaining the induced viral repression and the direction of virus evolution. This approach provided a general silencing strategy for limiting PRRSV replication by endogenous miRNAs in MARC-145 cells.

## Introduction

MicroRNAs (miRNAs) are the most important class of small [~18–24 nucleotides] noncoding RNAs and play a fundamental role in virus-host interactions [1]. The commonly accepted mechanism of miRNA regulation involves the miRNA seed region (~2–8 nucleotides at the 5′ end) binding to complementary sequences in the untranslated regions (UTRs) of target mRNAs, leading to gene regulation by reducing mRNA translation or transcript destabilization [2, 3]. This mechanism of miRNA regulation provides an opportunity to engineer viral genomes to include perfectly complementary miRNA-target sequences in an effort to transform host endogenous miRNA essentially intointerfering RNA and also has the potential to regulate the tissue tropism of viruses *in vivo* [4–6]. Based on this approach, Perez et al. generated an attenuated influenza A virus by incorporating nonavian miRNA response elements into the open-reading frame (ORF) of the viral nucleoprotein, which might be combined with existing live, attenuated influenza vaccines to increase attenuation and improve vaccine safety [7].

Porcine reproductive and respiratory syndrome (PRRS) is involved in reproductive failure in pregnant sows and respiratory illness particularly in young pigs [8]. PRRS is considered among the most severe infectious diseases threatening the swine industry worldwide, with PRRS-associated costs in millions of dollars annually and for which effective control measures remain scant [9]. PRRS virus (PRRSV), as a member of the family *Arteriviridae*, is an enveloped, single-stranded, positive RNA virus [10, 11].

In recent years, there has been an explosion in research associated with host miRNA-mediated gene regulation involving PRRSV pathogenesis. Several studies generated small RNA expression profiles to identify alterations in miRNA expression in porcine alveolar macrophages (PAMs) [12, 13]. Zhou et al. selected fourteen miRNAs to detect their abundance change during PRRSV infection in MARC-145 cells [14]. Several miRNAs were identified as binding directly to PRRSV genomic RNA, subsequently reducing viral infectivity and replication [15, 16]. Recent studies also demonstrated that modulation of specific miRNA expression (e.g., miR-125b, miR-26a, miR-24-3p, miR-30c, let-7f-5p, and miR-373) affects the development of the host response to viral infections [17–22]. In summary, miRNAs have exerted significant effects on viral-genome evolution and RNAi-mediated anti-PRRSV therapeutic strategies.

Despite the potential successes associated with miR-PRRSV interactions, few attempts have been made to engineer PRRSVs to interact with host endogenous miRNA pathways. We wonder if host endogenous miRNAs can be used to regulate the replication of PRRSV in an effort to control viral replication, generate a range of biological tools, or develop attenuated virus vaccines in the future. The full-length cDNA of the infectious PRRSV clone in our lab offers an opportunity to explore this strategy on PRRSV [23].

In this study, we identified the cellular miRNAome profiles from *in vitro*-cultured PRRSV-infected MARC-145 cells and manipulated the viability and replication of PRRSV using host endogenous miRNAs. Results showed that the copy number of endogenous miRNAs and the extent of small-RNA (sRNA) complementarity were two most important factors and the mutant containing miR-140-target sites exhibited strongly suppression of viral replication. This represents the first PRRSV-specific report on cloning miRNA-recognition sequences into the noncoding regions of viral genome.

## Materials and methods

### Cells and plasmids

MARC-145 cells were grown in minimal essential medium (MEM) (Sigma-Aldrich, St. Louis, MO, USA) supplemented with 10% fetal bovine serum (FBS) (Invitrogen, Carlsbad, CA, USA) and maintained in MEM supplemented with 2% FBS at 37°C with 5% CO_2_ as described previously [23]. Type 2 PRRSV infectious cDNA clone pAPRRS [GenBank accession number: GQ330474; [23]] was used as the wild-type (wt) controland p7USC was constructed by inserting an *Asc*I restriction site immediately after the stop codon of ORF 7 [24]. vAPRRS was rescued from pAPRRS, and high-titer virus stocks were obtained by infecting MARC-145 cells at an multiplicity of infection (MOIs) of 0.01. Infected cell supernatants were harvested when about 80% cells developed CPE, followed by storage at −80°C as stocks for further use. Viral titer was determined by standard 50% tissue culture infectious dose (TCID_50_) assay using MARC-145 cells [25].

### Illumina sRNA sequencing

A total of 10^7^ MARC-145 cells/plate were maintained for 24 h at 37°C with 5% CO_2_, followed by either mock infection or infection with PRRSV strain vAPRRS at an MOI of one in triplicate and incubation at 37°C for 1 h. Cells were washed with 1× phosphate-buffered saline (PBS), followed by the addition of fresh medium and then maintained at 37°C with 5% CO_2_. Cells were harvested in TRIzol reagent (Invitrogen) at 24 h post-infection. Triplicate plates were pooled, and total RNA was purified according to manufacturer’s instructions, with the exception of RNA samples that were separately precipitated overnight at −80°C. Genomic DNA was removed using the RNase-Free DNase Set (QIAGEN). RNA was quantified using a nanodrop ND-1000 spectrophotometer (Thermo Fisher Scientific, Waltham, MA, USA), and RNA quality was assessed by agarose gel electrophoresis. sRNA sequencing was further performed using Illumina HiSeq2000 (Illumina; Huada Genomics Institute Co., Ltd., China).

### Data analysis

For miRNA data analysis, raw-sequencing data were filtered for composition, the presence of adaptor dimers, length, sequence repetition, and copy numbers. The filtered data were then mapped to the known miRNAs of all organisms listed in the current miRBase (http://www.mirbase.org) and the *Macaca mulatta* genome. All miRNA-sequencing reads were sorted according to the barcode index, and adapter sequences were trimmed. Only high-quality reads (overall Phred ≥20) were selected. Identical sequences in each library were grouped using the GALAXY bioinformatics suite (https://main.g2.bx.psu.edu/) according to known *M. mulatta* miRNAs and homologous miRNAs from other species not yet present in the *M*. *mulatta* database in miRBase (http://www.mirbase.org).

### Candidate miRNAs

According to sRNA-expression profiles, six candidate cellular miRNAs were chosen to represent different miRNA-expression levels in PRRSV-infected MARC-145 cells. They included mml-miR-21, mml-miR-140-3p, mml-miR-185, mml-miR-26a, mml-miR-505, and mml-miR-199a as representatives of high-, moderate-, and low-abundant miRNAs. Their reverse-complementary sequences were inserted into PRRSV 3′UTRs, which artificially engineered the viral genome to contain complementary base-pairing-target sites for the corresponding miRNAs. Sequences (5′ to 3′), reverse-complementary sequences (5′ to 3′), and reads of the candidate miRNAs are listed in **Table 1**.

**Table 1:** Candidate miRNAs and reverse-complementary sequences.

miRNA	Sequence	Reverse Complementary Sequences	Reads
miR-21	UAGCUUAUCAGACUGAUGUU	AACAUCAGUCUGAUAAGCUA	2,369,620
miR-140	UACCACAGGGUAGAACCACGG	CCGUGGUUCUACCCUGUGGUA	539,458
miR-185	UGGAGAGAAAGGCAGUU CCUGA	UCAGGAACUGCCUUUCUCUCCA	19,621
miR-26a	UUCAAGUAAUCCAGGAUAGGCU	AGCCUAUCCUGGAUUACUUGAA	9,789
miR-505	CGUCAACACUUGCUGGUUUCCU	AGGAAACCAGCAAGUGUUGACG	127
miR-199a	CCCAGUGUUCAGACUACCUGUUC	GAACAGGUAGUCUGAACACUGGG	55

### Quantitative real-time PCR (qRT-PCR)

Assays to quantify mature miRNAs were conducted as previously described [26]. Briefly, 10 ng of total RNA was reverse transcribed to cDNA using the PrimeScript first-strand cDNA synthesis kit (Takara, Dalian, China) using specific stem-loop RT primers (**Table 2**). Samples were incubated at 42°C for 1 h and at 70°C for 5 min, followed by PCR for each miRNA using the transcription product, the miRNA-specific forward primer, and a universal reverse primer (UR) (**Table 2**). Real-time PCR was performed using a Step-one Plus real-time PCR system (Applied Biosystems, Foster City, CA, USA) and a SYBR Premix Ex Taq polymerase (Takara). In each assay, 2 μL cDNA was used for amplification according to the following conditions: 95°C for 1 min, followed by 40 cycles at 95°C for 15 s and 64°C for 30 s. MiRNA expression was normalized to U6 small nuclear RNA (snRNA). The relative quantitative miRNA-expression level was evaluated using the comparative Ct method [26]. Relative expression was calculated by the comparative ΔΔCt method, and the values were expressed as 2^−ΔΔCt^. Relative expression levels of viral ORF7 RNA were also analyzed using the ΔΔCt method, and glyceraldehyde-3-phosphate dehydrogenase (GAPDH) mRNA was used as an endogenous control. All qPCR experiments were performed in triplicate. Other primers are listed **Table 2.**

**Table 2:** Primers used to determine the relative expression level of host miRNAs by stem-loop qRT-qPCR and viral ORF7 RNA.

Primer	Sequence (5′−3′)
RT-miR-21	TGTCGGGTAGCTTATCAGACTGATGTTGACTGTTGAATCTCATGGCAACACCAGTCGATGGGCTGTCTGACAAACATCAG
RT-miR-140	UGUGUCUCUCUCUGUGUCCUGCCAGUGGUUUUACCCUAUGGUAGGUUACGUCAUGCUGUUCUACCACAGGGUAGAACCACGGACAGGAUACCGGGGCACCCCGTGGTT
RT-miR-185	AGGGCGCGAGGGATTGGAGAGAAAGGCAGTTCCTGATGGTCCCCTCCTCAGGGGCTGGCTTTCCTCTGGTCCTTCCCTCCCATCAGGAAC
RT-miR-26a	GTGGCCTCGTTCAAGTAATCCAGGATAGGCTGTGCAGGTCCCAATGGGCCTATTCTTGGTTACTTGCACGGGGACGCAGCCTATC
RT-miR-505	GATGCACCCAGTGGGGGAGCCAGGAAGTATTGATGTTTCTGCCAGTTTAGCGTCAACACTTGCTGGTTTCCTCTCTGGAGCATCAGGAAACC
RT-miR-199a	AGGAAGCUUCUGGAGAUCCUGCUCCGUCGCCCCAGUGUUCAGACUACCUGUUCAGGACAAUGCCGUUGUACAGUAGUCUGCACAUUGGUUAGACUGGGCAAGGGAGAGCAGAACAGGT
Q-miR-21(F)	TAGCTTATCAGACTGATGTTGAT
Q- miR-140 (F)	GGGGTACCACAGGGTAGAAC
Q-miR-185(F)	GGTGGAGAGAAAGGCAGTTCCTGA
Q-miR-26a (F)	TTCAAGTAATCCAGGATAGGCTG
Q-miR-505 (F)	CCCGTCAACACTTGCTGGTTTCC
Q-miR-199a (F)	ACACTCCAGCTGGGCCCAGTGTTCAGACT
UR	CAGTGCGTGTCGTGGAGT
U6-F	CTCGCTTCGGCAGCACA
U6-R	AACGCTTCACGAATTTGCGT
ORF7-F	CCCTAGTGAGCGGCAATTGT
ORF7-R	TCCAGCGCCCTGATTGAA
GAPDH-F	CCTTCCGTGTCCCTACTGCCAAC
GAPDH-R	GACGCCTGCTTCACCACCTTCT

### Mutagenesis of PRRSV 3′ UTRs containing candidate miRNA-target sites

For the construction of plasmids containing primary engineered insertion mutations, appropriate fragments were prepared by RT-PCR products from the viral cell-culture supernatants. The amplified fragments between the *Asc*I and *Xho*I restriction sites were ligated into the corresponding regions of pAPRRS. All full-length mutant clones were verified by *Sma* I mapping and nucleotide sequencing. The primers used for PCR to construct mutant-viruses are shown in **Table 3.** “SF” in the designations represents upstream primer, whereas “Qst” was used as downstream primer. SF14841 and SR15497 were used for DNA sequencing.

**Table 3:** Primers used to construct mutant PRRSVs harboring miRNA target sites.

Primer	Sequence (5′–3′)
SF505-199-t	TT*GGCGCGCC***AGGAAACCAGCAAGTGTTGACG GAACAGGTAGTCTGAACACTGGG** TGGGCTGGCATTCTTGAGGC
SF505-199-c	TT*GGCGCGCC***AGCATACTATCGAGAGTTGCCG GACCATGTCGCCTTAAGACTGCG** TGGGCTGGCATTCTTGAGGC
SF185-26-t	TT*GGCGCGCC***TCAGGAACTGCCTTTCTCTCCA AGCCTATCCTGGATTACTTGAA** TGGGCTGGCATTCTTGAGGC
SF185-26-c	TT*GGCGCGCC***TCAGCATCAGGCTTTGTCACGA ATCCTCTCGTGGAATAGTAGTA** TGGGCTGGCATTCTTGAGGC
SF21-t	TT*GGCGCGCC***AACATCAGTCTGATAAGCTA** TGGGCTGGCATTCTTGAGGC
SF21-c	TT*GGCGCGCC***TACGTCTGCCTCACAATCTA** TGGGCTGGCATTCTTGAGGC
SF140-t	TT*GGCGCGCC***CCGTGGTTCTACCCTGTGGTA** TGGGCTGGCATTCTTGAGGC
SF140-c	TT*GGCGCGCC***CGGTGCTACTAGCCAGTCGAA** TGGGCTGGCATTCTTGAGGC
SF21-140-t	TT*GGCGCGCC***AACATCAGTCTGATAAGCTA CCGTGGTTCTACCCTGTGGTA** TGGGCTGGCATTCTTGAGGC
SF21-140-7c	TT*GGCGCGCC***TACGTCTGCCTCACAATCTA CGGTGCTACTAGCCAGTCGAA** TGGGCTGGCATTCTTGAGGC
SF21-140-5c	TT*GGCGCGCC***AACGTCTGCCTCACAAGCTA CCGTGCTACTACCCAGTCGAA** TGGGCTGGCATTCTTGAGGC
SF21-140-3c	TT*GGCGCGCC***AACATCTGCCTCATAAGCTA CCGTGCTACTACCCTGTGGAA** TGGGCTGGCATTCTTGAGGC
SF21-140-1c	TT*GGCGCGCC***AACATCAGTCTCATAAGCTA CCGTGGTACTACCCTGTGGTA** TGGGCTGGCATTCTTGAGGC
Qst	GAGTGACGAGGACTCGAGCGCATGCTTTTTTTTTTTTTT
SF14841	GCAGGCTTTCATCCGATTA
SR15497	CAAT TAAATCTTACCCCCACAC

### Transfection and rescue of mutant viruses

Full-length plasmids were prepared using a QIAprep spin miniprep kit (QIAGEN). MARC-145 cells were seeded into six-well plates and grown to 80% confluence. The monolayer cells were transfected with 2 μg plasmid using 5 μL FuGENE HD reagents (Promega, Madison, WI, USA) according to manufacturer’s instructions. The wt and recovered mutant viruses were passaged in MARC-145 cells five times to gain P1–P5 virus stocks. At least three additional passages were performed when no visible CPE was observed after transfection. For analysis of viral growth kinetics, supernatants (0.2 mL/well) from cell cultures were collected at different time point post-transfection and frozen at −80°C. For virus quantification at each time point, a viral titer was measured in MARC-145 cells by standard TCID_50_ assay using a previously described method [25].

### Indirect immunofluorescence assay (IFA)

MARC-145 cells were transfected with wt or mutant plasmids, and intracellular expression of viral N proteins were visualized by immunofluorescence staining at different time points post-transfection, respectively, according to a protocol described previously [27]. Cells were fixed with cold methanol, followed by blocking with 1% bovine serum albumin and incubation for 2 h with a monoclonal antibody (SR30A; Rural Technologies, INC) specifically recognizing viral nucleocapsid (N) protein. After washing with PBS, the cells were incubated for 1 h with an Alexa Fluor 568-labeled goat anti-mouse secondary antibody (Invitrogen). After a final PBS wash step, cells were visually analyzed using an Olympus inverted fluorescence microscope (Olympus, Tokyo, Japan).

### Viral plaque assay

To purify rescued viruses, the supernatants from MARC-145 cells transfected with p140-t were serially 10-fold diluted in MEM and then were inoculated onto MARC-145 monolayers in six-well culture plates. After 1 h at 37°C, the monolayers were washed twice with PBS and overlaid with 2 mL MEM containing 1% (w/v) low melting-point agarose and 2% FBS, and then incubated for a further 96 h at 37°C. To analyze the plaque size of v140-t from different passages, MARC-145 monolayers were infected with v7USC, v140-t P1, v140-t P3, v140-t P5, and v140-t P10, overlaid with low melting-point agarose and stained with 5% (w/v) crystal violet in 20% ethanol at 5 dpi [28].

### Transfection of miRNA mimics and inhibitors

MiR-140-3p mimic and inhibitor (140-3p-inhi) were synthesized by GenePharma (Shanghai, China) as double-stranded 2′-O-methyl-modifed RNA oligonucleotides. The negative-control (NC) mimic sequence was 5′-uucuccgaacgugucacgutt-3′ and NC inhibitor (NC-inhi) sequence was 5′-caguacuuuuguguaguacaa-3′. MiRNA or NC mimics were transfected into MARC-145 cells at a concentration of 80 nM using X-tremeGENE siRNA Transfection Reagent (Roche). Twenty-four hours after transfection, cells were infected with PRRSV. For virus quantification at each time point, a viral titer was measured in MARC-145 cells by standard TCID_50_ assay using the method of Reed and Muench [25].

### Nucleotide sequencing

Viral RNAs (P1–P10) were extracted from cell supernatants using a QIAamp viral RNA kit (Qiagen) and treated with TURBO DNase (Ambion; Thermo Fisher Scientific) to eliminate input genomic DNA. First-strand cDNA was synthesized using the antisense primer Qst (**Table 3**). The fragment containing the 3′ UTR was amplified by Taq DNA polymerase (Takara) using primer pairs SF14841 and SR15497 (**Table 3**). The full-length genome of the recovered viruses was amplified using five primer pairs as described previously [23]. The primer sequences are available upon request.

## Results

### Characteristics of the sRNA libraries

**Fig 1:**
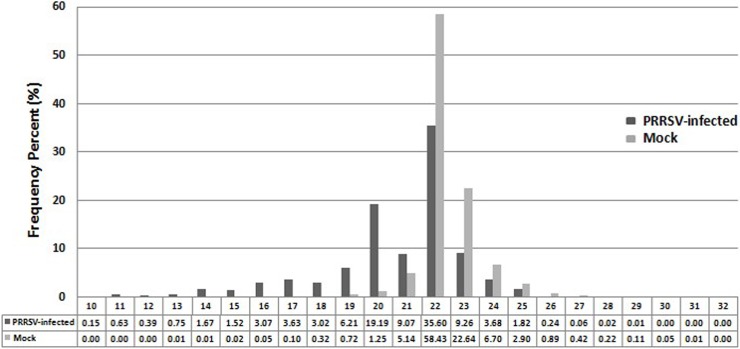
Length distributions of sRNAs (10–32 nucleotides) in PRRSV-infected and uninfected MARC-145 cells. **s**RNA libraries from PRRSV-infected MARC-145 cells *in vitro* were analyzed using Illumina deep sequencing. Within each sample, 86.03% and 95.2% high-quality sRNAs were ~18 to 24 nucleotides in length, with most sRNAs 22 nucleotides in length.

To investigate the miRNAome of MARC-145 cells and the effect of PRRSV infection on miRNA expression, sRNA libraries from PRRSV-infected MARC-145 cells *in vitro* were analyzed using Illumina deep sequencing. A total of 8,382,351 and 16,433,979 sRNA reads of 10 to 35 nucleotides in length were obtained from mock- and PRRSV-infected MARC-145 cells, respectively. After removing low-quality reads and masking adaptor sequences, 8,345,223 (97.62%) and 14,906,801 (94.03%) clean sRNA reads were obtained from the two sRNA libraries, respectively. Within each sample, 86.03% and 95.2% high-quality sRNAs were from 18 to 24 nucleotides in length, with most 22 nucleotides in length **(Fig 1)**. Ultimately, 5,955,834 and 9,636,250 miRNA reads from the two libraries were matched to known host miRNA sequences. Read numbers of all known miRNAs were listed in S1 **Table**. The 30 most commonly sequenced miRNAs in two samples are listed in **Table 4**. The most highly expressed miRNA in PRRSV-infected samples was mml-miR-21, representing ~25% of the total miRNA reads **(Table 4)**. By mapping the clean reads to miRBase, we detected 260 known miRNAs in two libraries while the 30 most abundant miRNAs accounted for 97.5% and 95.2% of the total miRNA reads in mock- and PRRSV-infected samples, respectively **(Table 4)**. Among the 30 most abundant miRNAs, the most strongly expressed miRNA family in both libraries was mml-let-7 (let-7a, 7b, 7c, 7d, 7e, 7f, 7g, and 7i). This was consistent with a previous study reporting the let-7 family is highly expressed in various cell types and species [29].

**Table 4:** The thirty most commonly sequenced miRNAs in PRRSV-infected and uninfected MARC-145 cells.

Ranking	PRRSV-infected		Mock	
	miRNA	Reads	miRNA	Reads
**1**	**mml-miR-21**	**2,369,620**	**mml-let-7a**	**1,891,264**
**2**	**mml-let-7a**	**1,865,115**	**mml-let-7f**	**1,267,281**
**3**	**mml-let-7f**	**1,569,627**	**mml-let-7b**	**685,399**
**4**	**mml-miR-140-3p**	**539,458**	**mml-let-7e**	**399,487**
**5**	**mml-miR-103**	**474,309**	**mml-miR-140-3p**	**239,031**
**6**	**mml-miR-107**	**338,159**	**mml-let-7d**	**194,210**
**7**	**mml-let-7e**	**279,199**	**mml-miR-423-5p**	**156,306**
**8**	**mml-miR-221**	**199,587**	**mml-miR-103**	**129,878**
**9**	**mml-miR-30a-5p**	**194,948**	**mml-miR-320a**	**125,980**
**10**	**mml-let-7b**	**179,225**	**mml-miR-181a-5p**	**105,682**
**11**	**mml-miR-320a**	**168,605**	**mml-miR-21**	**86,272**
**12**	**mml-miR-101**	**134,344**	**mml-miR-181b**	**69,008**
**13**	**mml-miR-191**	**99,683**	**mml-miR-221**	**52,644**
**14**	**mml-miR-222**	**96,944**	**mml-let-7g**	**46,468**
**15**	**mml-let-7i**	**83,662**	**mml-let-7c**	**44,881**
**16**	**mml-let-7d**	**71,672**	**mml-miR-29a**	**43,775**
**17**	**mml-miR-99b**	**69,402**	**mml-miR-191**	**39,192**
**18**	**mml-miR-378a**	**62,518**	**mml-miR-107**	**36,457**
**19**	**mml-miR-29a**	**58,790**	**mml-miR-25**	**32,751**
**20**	**mml-miR-10a**	**51,953**	**mml-miR-222**	**31,316**
**21**	**mml-miR-181a-5p**	**42,287**	**mml-let-7i**	**24,839**
**22**	**mml-miR-125a-5p**	**38,676**	**mml-miR-23a**	**15,398**
**23**	**mml-miR-34c-5p**	**34,325**	**mml-miR-24-3p**	**15,177**
**24**	**mml-miR-192**	**32,952**	**mml-miR-30a-5p**	**12,415**
**25**	**mml-miR-92a**	**28,223**	**mml-miR-378a**	**12,034**
**26**	**mml-miR-423-5p**	**27,857**	**mml-miR-34c-5p**	**11,035**
**27**	**mml-miR-23a**	**22,243**	**mml-miR-30d**	**10,173**
**28**	**mml-miR-185**	**19,621**	**mml-miR-128a**	**9,956**
**29**	**mml-miR-25**	**11392**	**mml-miR-185**	**9,771**
**30**	**mml-miR-26a**	**9,789**	**mml-miR-99b**	**9,169**

### Validation of the abundance of candidate miRNAs

**Fig 2:**
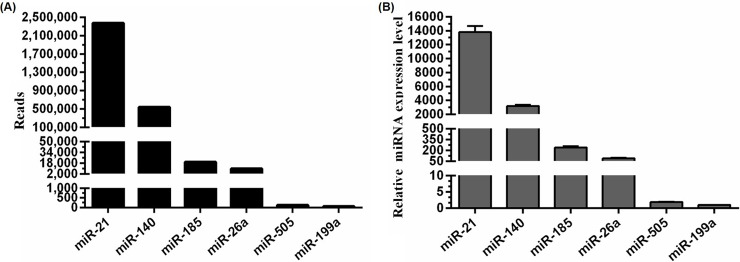
Copy numbers of candidate miRNAs determined by Illumina deep sequencing and validation of their expression levels using stem-loop qRT-PCR. **A:** Reads of the six selected miRNAs obtained from PRRSV-infected MARC-145 cells are shown along the Y-axis (*black bars*). **B:** MARC-145 cells were infected with vAPRRS at an MOI of one, and miRNA expression was determined at 24 hpi using stem-loop qRT-PCR. Relative expression levels were normalized against snRNA U6. Data are shown as the mean ± standard deviation (SD) of three independent experiments.

According to deep-sequencing results, mml-miR-21 was the most highly expressed miRNA (2,369,620 reads) in PRRSV-infected samples, whereas mml-miR-505 and mml-miR-199a returned relatively few reads (127 and 55, respectively) and represented the low-abundant miRNAs, whereas mml-miR-185 and mml-miR-26a (19,621 and 9,789 reads, respectively, were moderate-abundant miRNAs (**Fig 2A**). To validate these results, stem-loop qRT-PCR was performed to detect the relative expression levels of endogenous candidate miRNAs in PRRSV-infected samples. Our results showed that the relative expression of mml-miR-21 was ~5-fold higher than that of mml-miR-140, ~150-fold higher than that of mml-miR-26a, and ~13,500-fold higher than that of mml-miR-199a (**Fig 2B**). These results were consistent with their abundance as determined by deep sequencing.

### Generation of mutants harboring miRNA-target sequences

**Fig 3:**
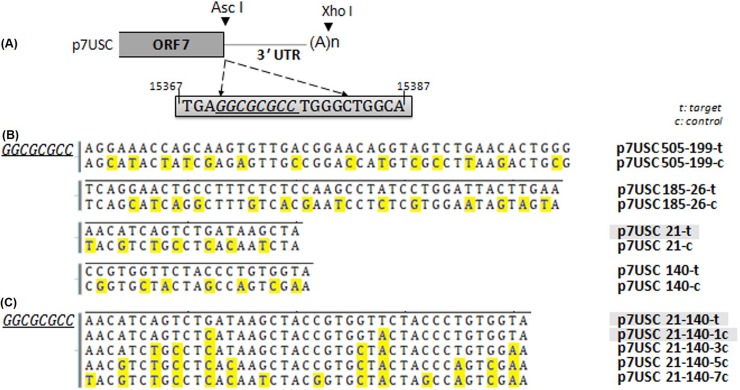
Schematic diagram of PRRSV mutants containing miRNA-target sites. **A:** Schematic drawing of the insertion sites in the viral genome. The gray box indicates part of the viral genome. p7USC is a full-length infectious cDNA clone containing an *Asc*I site located after the stop codon of ORF7. **B** and **C:** Construction strategy of miRNA-target sites inserted into mutants. Residues in yellow shade indicate the mutated sites as controls.

We used a recombinant full-length clone (p7USC) containing an *Asc*I restriction site located immediately after the stop codon of ORF7 as the mutant vector. Schematic diagrams of mutant-virus construction are shown in **Fig 3A**. Transfection assays confirmed that insertion of the *Asc*I site did not affect viral infectivity, and that the rescued virus (7USC) showed similar growth properties as that of the wt virus (vAPRRS) [24]. Therefore, p7USC was used as the backbone for constructing mutants harboring inserted miRNA-target sites designated as follows: p505-199-t, p505-199-c, p185-26-t, p185-26-c, p140-t, p140-c, p21-t, p21-c, p21-140-t, p21-140-7c, p21-140-5c, p21-140-3c, and p21-140-1c (**Fig 3B and 3C**). The letter “t” in the designations represents their status as miRNA-target sequences, whereas the letter “c” represents their status as control mutants harboring several nucleotides mutations in the target sequences. All mutant plasmids were confirmed by sequencing.

### Blockage of viral transcription and replication by the most highly abundant miRNA-target mutant

**Fig 4:**
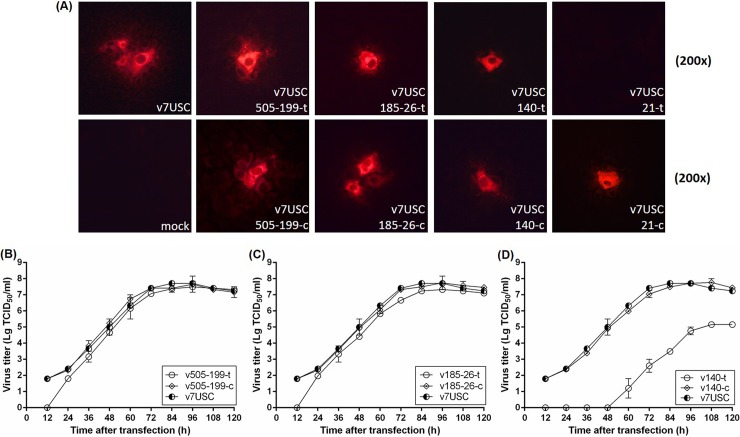
Mutants harboring sequences targeted by miRNAs of different abundant levels affect viral replication. **Immunofluorescence staining for intracellular N protein in mutants. A:** Confluent MARC-145 cells were transfected with plasmids p505-199-t, p505-199-c, p185-26-t, p185-26-c, p140-t, p140-c, p21-t, and p21-c, as indicated respectively, with p7USC and mock-transfected cells used as controls. Cells were fixed at 24 hpt and immunostained with the mouse monoclonal SR30A antibody against the viral N protein and fluorescein isothiocyanate-conjugated goat anti-mouse IgG. **B:** Viral growth after transfection of p7USC, p185-26-t, or p185-26-c, as representatives of moderate-abundant miRNAs. **C:** Viral growth after transfection of p7USC, p140-t, and p140-c, as representative of high-abundant miRNAs. **D:** Viral growth after transfection of p7USC, p21-140-7c, p21-140-5c, and p21-140-3c, as representatives of mutant controls of high-abundant miRNA. Viral titers were expressed as Lg TCID_50_/mL. Data are shown as the mean ± SD of three independent experiments.

To determine whether mutants harboring sequences targeted by miRNAs of different abundant levels affect viral replication, full-length clones p505-199-t, p505-199-c, p185-26-t, p185-26-c, p140-t, p140-c, p21-t, and p21-c were transfected into MARC-145 cells, and intracellular expression of the viral N protein was determined by IFA at 24 h post-transfection (hpt) to evaluate genomic RNA replication and subgenomic mRNA transcription in the mutants. As shown in **Fig 4A**, all mutants, except that p21-t, displayed N protein expression. The fluorescence intensity **(Fig 4A)** and viral growth properties after transfection **(Fig 4B and 4C)** of the p505-199-t and p185-26-t mutants were consistent with those observed in p7USC and their control mutants (p505-199-c and p185-26-c, respectively), although the viral titers of p505-199-t and p185-26-t were slightly lower than those of the wt virus at 12 hpt **(Fig 4B and 4C)**, suggesting that mutants containing low- and moderate-abundant miRNA-target sites exhibited similar transcription and replication properties as those of the wt virus. After transfection of p140-t into MARC-145 cells, fluorescent signals associated with N-protein expression were detected at 24 hpt (**Fig 4A**), but no infectious particle were detected at 48 hpt (**Fig 4D**). Moreover, no fluorescent signal was detected in the p21-t mutant, even after a prolonged incubation (7-days post-transfection) (data not shown), indicating that mml-miR-21, which was the most highly abundant miRNA detected in PRRSV-infected MARC-145 cells, blocked both viral replication and transcription. Supernatant (1 mL/well) from cell cultures transfected with p21-t were collected and underwent at least three additional passages, after which no visible CPE was observed, indicating that inserting of miR-21-target site inter the viral genome abolished PRRSV viability and replication.

### Discontinuous mutations in target sites associated with highly abundant miRNA recovered viral viability and propagation

**Fig 5:**
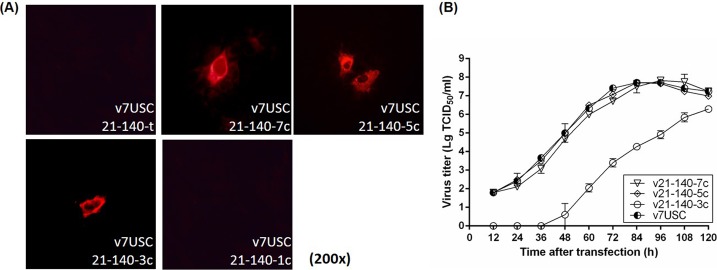
Discontinuous mutations in target sites recovered viral viability and propagation. **A:** Intracellular viral N-protein expression of high-abundance miRNA-target mutants and control plasmids (p21-140-t, p21-140-7c, p21-140-5c, p21-140-3c, and p21-140-1c). **B:** Confluent MARC-145 cells were transfected with low-abundant miRNA mutants and control plasmids (p7USC, p505-199-t, and p505-199-c). Supernatants were collected at the indicated times after transfection and titrated for viral-titer determination. Data are shown as the mean ± SD of three independent experiments.

We then determined the relationships between differences in viral replication and the inserted miRNA-target sites. Combination of the miR-21- and miR-140-target sequences (p21-140-t), followed by inserting into p7USC, resulted in no fluorescent signal, and no infectious particles were detected in the case of p21-140-t, even after three additional passages (**Fig 5A**). Point mutation of a single nucleotide in two miRNA-target sequences (p21-140-1c), respectively, continued to return no fluorescent signal and generated no infectious particles (**Fig 5A**), whereas point mutation of three nucleotides in the two miRNA-target sequences (p21-140-3c), respectively, resulted in a fluorescent signal associated with N-protein expression at 24 hpt (**Fig 5A**), and suppression of viral growth after transfection (**Fig 5B**). Point mutations of five or seven nucleotides (p21-140-5c and p21-140-7c), respectively, resulted in fluorescent signals and viral-growth patterns after transfection similar to those of v7USC (**Fig 5B**). These results suggested that nucleotide mutations in complementary miRNA-target sequences determined the efficiency of endogenous miRNA binding and subsequently influenced virus viability and replication.

### Mutants harboring miR-140-target sequences (v140-t) exhibit strongly suppressed viral replication

**Fig 6:**
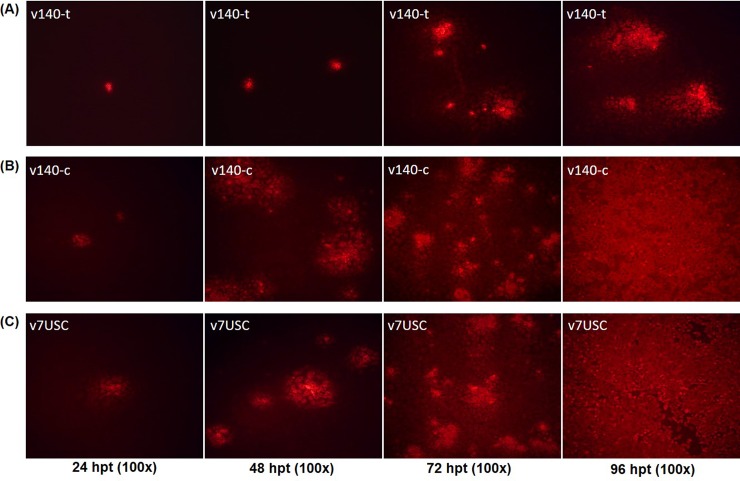
Virological characteristics of mutants. Intracellular N-protein expression of **A**: v140-t and **B:** v140-c at the indicated times (24 hpt, 48 hpt, 72 hpt, and 96 hpt), with **C:** v7USC was used a control. Cells were fixed and immunostained with the mouse monoclonal SR30A antibody against the viral N protein and fluorescein isothiocyanate-conjugated goat anti-mouse IgG.

The relative expression of endogenous miR-140 was ~5-fold lower than that of miR-21 according to stem-loop qRT-qPCR (**Fig 3B**), making it the fourth highest expressed miRNA detected in PRRSV-infected samples. Notably, the fluorescence intensity of the v140-t mutant differed from that of the control mutant (v140-c) and wt v7USC at the indicated times (24 hpt, 48 hpt, 72 hpt, and 96 hpt) **(Fig 6)**. Staining revealed only a few single cells positive for v140-t at 24 hpt and 48 hpt **(Fig 6A)**, suggesting that viral transcription and replication were strongly suppressed in this mutant. However, at 96 hpt, almost all cells associated with the control mutant (v140-c) and v7USC exhibited positive staining **(Fig 6B and 6C)**, whereas minimal fluorescence intensity was observed from v140-t cells **(Fig 6A)**, indicating that mutants harboring miR-140-target sites exhibited strong suppression of viral replication following transfection. The viral-growth properties of the v140-t mutants after transfection were consistent with the fluorescence-staining results. As shown in **Fig 4D**, compared with v140-c, viral titers of v140-t were 10^4^-fold lower during the time period from 60 hpt to 84 hpt and 10^3^-fold lower from 96 hpt to 120 hpt, whereas v140-c maintained similar growth properties as those observed in v7USC.

**Fig 7:**
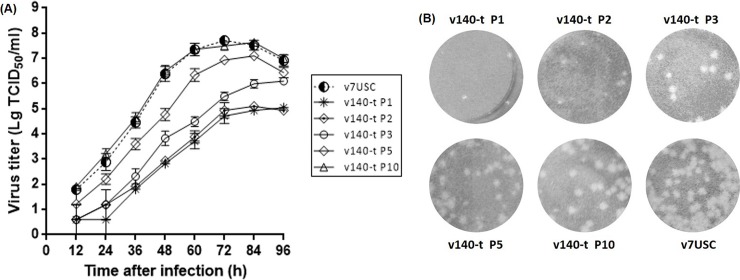
Viral growth and plaques characteristics of v140-t. **A:** Analysis of v140-t (P1, P3, P5 and P10) growth in MARC-145 cells. Culture supernatants were collected at the indicated times and titrated. **B:** Analysis v140-t (P1, P3, P5 and P10) plaques characteristics in MARC-145 cells. MARC-145 monolayers were infected with v7USC, v140-t P1, v140-t P3, v140-t P5, and v140-t P10, respectively, overlaid with low melting-point agarose and stained with 5% (w/v) crystal violet in 20% ethanol at 5 dpi.

Through purifing rescued v140-t by plaque assay, we harvested the correct sequencing virus v140-t P1. MARC-145 cells were infected with the virus at an MOI of 0.01, and culture supernatants were collected as viral stocks at P2 to P10. Viral stocks (P1, P2, P3, P5, and P10) were used in the growth kinetics and plaque morphology experiments. Results showed that the peak titer of v140-t increased by passages. The peak titer was significant lower for v140t P1-P3 than for v7USC. Viral propagation of v140-t P5 increased in late hours post infection and v140-t P10 displayed almost the same virological properties with v7USC (**Fig 7A**). When compared with the wt virus, v140-t P1-P3 were distinguishable based on the size and morphology of viral plaques. As shown in **Fig 7B**, the plaque sizes of v140-t P1-P3 were smaller than those of the v7USC, whereas the v140-t P5 generated plaques with a mixture of different sizes and v140-t P10 exhibited similar behavior to the wt, indicating that v140-t showed strongly suppressed viral replication compared to wt virus in the early passages based on viral propagation and plaques characteristics.

### MiR-140-3p inhibits the mutant v140-t but not wt virus

**Fig 8:**
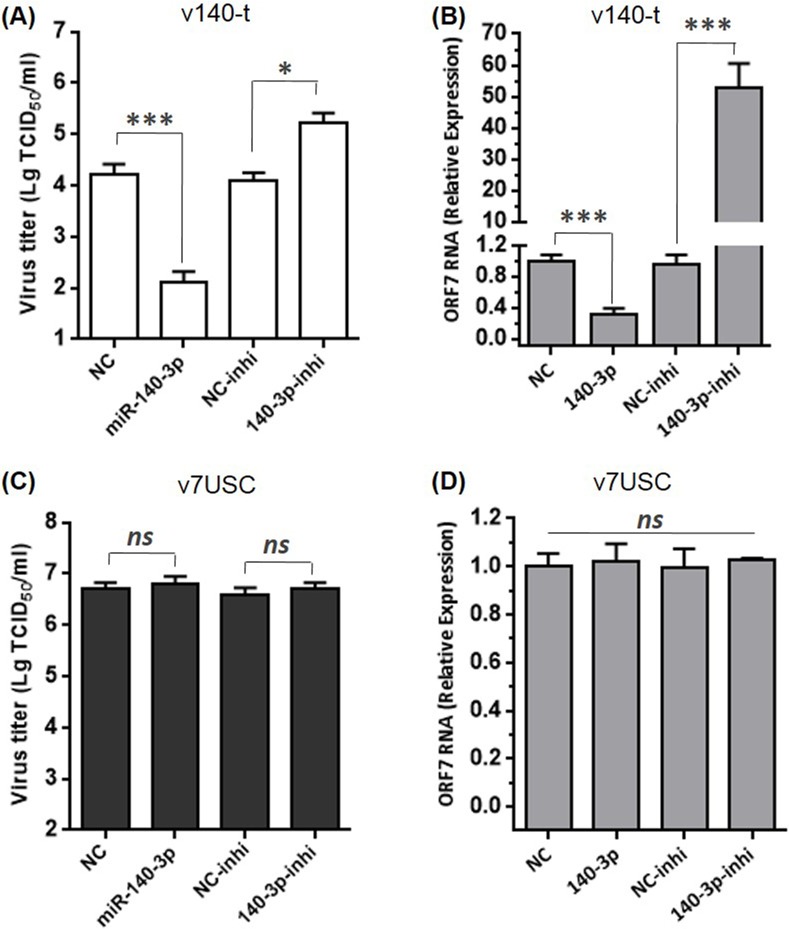
Over-expression of miR-140-3p inhibits the mutant v140-t but not wt virus. **A and C:** Confluent MARC-145 cells were transfected with the indicated miR-140-3p mimics, inhibitors, or NC. After 24 h, cells were infected with v140-t or v7USC (MOI = 0.05). The supernatants were collected at 36 h to determine viral titers. Virus titers were expressed as the lg TCID_50_/ml. **B and D:** qRT-PCR analysis of viral ORF7 RNA levels in MARC-145 cells transfected with miR-140-3p mimics, inhibitors, or NC, followed by v140-t or v7USC infection. The data were normalized to GAPDH expression.

In order to validate the effect of miR-140-3p to the viral repression of v140-t, we chose v140-t P3 and v7USC as the objects in this experiment. For v140-t P3, MARC-145 cells transfected with miR-140-3p mimics yielded significantly lower viral titers and ORF7 gene expression compared with cells transfected with the NC **(Fig 8A and 8B)**, while transfection of miR-140-3p inhibitors demonstrated the opposite effects **(Fig 8A and 8B)**, indicating that over-expression of miR-140-3p recognized the binding region in v-140t genome and further inhibited viral replication. However, for v7USC, over-expression of miR-140-3p or silencing had no impact on viral titers and ORF7 RNA level due to the absence of miR-140-target sequences in wt virus **(Fig 8C and 8D)**. These results verified that the abundance of miR-140-3p in MARC-145 cells played a decisive role in determining gene expression level and viral growth of v140-t.

### Viruses are capable of resisting host miRNA targeting through spontaneous mutation of the seed region

**Fig 9:**
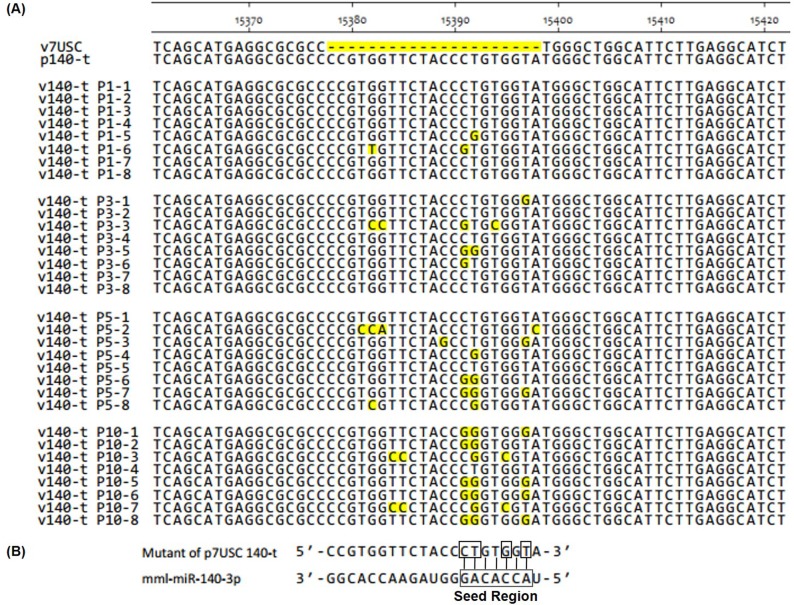
Genetic stability of v140-t (P1, P3, P5 and P10) according to nucleotide sequencing. **A:** To assess their genetic stability, the supernatants of v140-t (P1, P3, P5 and P10) passages were collected, and the cDNA fragment (nucleotides 14841–15497) containing the insertion sites were amplified by RT-PCR and cloned into the pMD-18T vector for transformation into *Escherichia coli*. Primer SF14841 was used to analyze the nucleotide sequences, and alignments were performed among the different clones of v140-t in P1, P3, P5, and P10. Spontaneous mutations were shaded in yellow. **B:** Schematic diagram of spontaneous mutations in the v140-t mutant and the seed region of mml-miR-140-3p.

To investigate why the peak titer of v140-t increased by passages, nucleotide-sequencing analysis was performed to confirm the retention of the engineered miRNA-target-sequence insertions in all of the passaged viruses. The genetic stability of all rescued viruses (v505-199-t, v505-199-c, v185-26-t, v185-26-c, v140-c, and v21-c), except v140-t, showed no mutation(s) in the engineered insertion regions at all passage levels for three independent transfection assays (data not shown). However, by passage P10, several spontaneous mutations in the engineered insertion regions of v140-t appeared as compared with the mutant plasmid p140-t (**Fig 9A**). The full-length sequence of v140-t showed no further mutations in other genomic regions. Another independent transfection experiment was performed to confirm the occurrence of these spontaneous mutations, resulting in observation of different spontaneous mutations between the two experiments. The spontaneously mutated nucleotides CT15391-15392GG occurring in the 3′ end of the insertion region **(Fig 9A)** were identical with those in mutant virus v140-t in experiment 1. Interestingly, analysis of v140-t revealed different spontaneous mutations preferentially located among 2–8 nucleotides near the 3′ end of the insertion region and corresponding to the “seed region” of mml-miR-140 **(Fig 9B),** indicating the preference of virus escape from the endogenous miRNA suppressive pressure.

## Discussion

Small-regulatory RNAs, specifically miRNAs, are attractive candidates as regulators of viral and host-cell gene expression due to their small size, lack of immunogenicity, and remarkable functional flexibility. In this study, we described a strategy on PRRSV for manipulating viral replication by exploiting the regulatory capacity of host endogenous miRNA gene-silencing machinery, demonstrated that the copy number of endogenous miRNAs and the extent of sRNA complementarity were key factors associated with their silencing capability and thereby determine PRRSV viability. Notably, the mutants harboring miR-140-target sequences (v140-t) exhibited strongly suppression of viral replication in early passages.

We first determined the consequences of PRRSV infection on cellular miRNA expression profiles (**Fig 1 and Table 4**). MARC-145 cells were used to analyze the characteristics of cellular miRNA expression in response to PRRSV infection, given its status as the primary target cell used for *in vitro* PRRSV replication [30]. To determine the importance of copy number for engineering PRRSV mutants, we selected candidate miRNAs for further study based on deep-sequencing results and highlighted the 30 miRNAs accounting for >95% of the total miRNA reads in mock- and PRRSV-infected samples **(Table 4)**, which was consistent with previous reports using other cell lines [12, 31, 32]. We chose different miRNAs exhibiting different expression levels representing high-, moderate-, and low-abundant miRNAs, respectively, subsequently verifying the reliability of these levels by stem-loop qRT-PCR (**Fig 2**).

Hicks et al. generated sRNA-expression profiles to study alterations in miRNAome from PRRSV-infected PAMs [12]. Consistent with those results, here, we found that miR-21 exhibited the highest expression in PRRSV-infected MARC-145 cells, representing ~25% of the total miRNA reads **(Table 4)**. As one of the earliest miRNAs discovered, miR-21 is a broadly conserved miR, with perfectly identical sequences conserved among mammals [33, 34]. Insertion of the miR-21-target sequence into the viral genome reduced viral viability and inhibited viral replication, whereas the control mutant (v21-c) retained the ability to propagate to a level similar with that of wt virus, indicating that miR-21 binding to its inserted target sequence in the viral RNA genome was lethal to PRRSV. As the fourth-highest expressed miRNA under viral infection, the relative expression of miR-140-3p was ~5-fold lower than that of miR-21 **(Fig 2)**. Unlike the miR-21-target mutant, the viability of v140-t mutants could be rescued, but viral replication was strongly suppressed, and no infectious particles were observed until 60 hpt **(Figs 4 and 6)**. Additionally, the targets of miRNAs exhibiting low- and/or moderate-abundant and their respective control mutants (v505-199-t, v505-199-c, v185-26-t, and v185-26-c) (**Fig 4**) showed similar growth properties as those of v7USC and maintained genetic stability across five additional passages. Considering that the relative expression of miR-26a and miR-199 was ~150- and 13,500-fold lower than that of miR-21 (**Fig 2**), we found low-copy numbers of endogenous miRNA were insufficient for silencing viral replication. Furthermore, our results indicated that few miRNAs were expressed at the concentrations required to be effective virus silencers, given that the most abundant 20 to 30 miRNAs expressed following viral infection consistently represented >90% of all miRNAs.

We then inserted random point mutations consisting of different numbers of nucleotides in the target sequences (v21-140-3c, v21-140-5c, and v21-140-7c) **(Fig 3)**. The lack of contiguous base pairing between the mutated sequences and the miRNA prevented targeting and cleavage by the RNA-induced silencing complex [35], thereby promoting viral viability and propagation **(Fig 5)** and demonstrating that the extent of sRNA complementarity determined the viability of the PRRSV mutants. Perfect sequence complementarity between the endogenous miRNA and the target sequence inserted into the PRRSV genome was required for endogenous miRNA to achieve regulatory capacity.

The engineered influenza A virus containg miRNA response elements in the ORF of the nucleoprotein showed a reduction in mortality compared with control viruses and elicited a diverse antibody response [7]. To avoid introduce the artificial amino acids to viral structure proteins, we chose the site in the 3’UTR of PRRSV, which was proved to be dispensable for viral viability in the previous study [36]. Results showed that the mutant containing miR-140-target sites strongly suppressed viral replication compared to wt virus in the early passages (P1-P3) based on viral propagation and plaques characteristics **(Figs 7 and 8)**. Analysis of the engineered insertions showed spontaneous and identical mutation of nucleotides CT15391-15392GG in the 3′ end of the insertion region **(Fig 9)** by passages. Interestingly, different spontaneous mutations preferentially localized among 2–8 nucleotides near the 3′ end of the insertion region, which corresponds to the “seed region” of mml-miR-140. Similar to results associated with Dengue virus, escape of miRNA-mediated silencing occurred; however, our results differed, in that the Dengue virus underwent complete excision of the target area rather than through single-nucleotide mutations [4, 37]. Our results suggested that binding of host miR-140-3p to the target sequence in the viral genome affected viral-genome evolution and restored PRRSV viability by promoting spontaneous mutations corresponding to the “seed region”. As a fast-evolving RNA virus [38], the relatively high mutation rate of PRRSV might limit the application of RNA-interference-mediated antiviral therapies [39].

Our findings demonstrated the efficacy of utilizing abundant host miRNAs to manipulate PRRSV viability and replication. This method requires a high copy number of endogenous miRNAs and perfect complementarity of the miRNA-target sequence inserted into the viral genome. Although virus escape from the endogenous miR-140-3p suppressive pressure occurred, v140-t showed strongly suppression in the early passages. This represents the first PRRSV-specific report on viral manipulation through the insertion of miRNA-recognition sites into the non-coding regions of the viral genome and provides valuable insight into strategies for engineering interactions between PRRSV and host miRNAs.

## Supporting information

S1 Table MiRNA-read numbers in PRRSV-infected and uninfected MARC-145 cells. (XLS)
